# Genome Sequence of a Goose Parvovirus Strain Isolated from an Ill Goose in China

**DOI:** 10.1128/genomeA.00227-17

**Published:** 2017-04-27

**Authors:** Peng Liu, Jinyue Zhang, Shun Chen, Mingshu Wang, Anchun Cheng

**Affiliations:** a Institute of Preventive Veterinary Medicine, Sichuan Agricultural University, Chengdu, Sichuan, China; b Research Center of Avian Disease, College of Veterinary Medicine of Sichuan Agricultural University, Chengdu, Sichuan, China; c Key Laboratory of Animal Disease and Human Health of Sichuan Province, Chengdu, Sichuan, China

## Abstract

We report here the complete genome sequence of a goose parvovirus (GPV) strain, RC16, which was isolated from an ill goose in 2016 at Rongchang, Chongqing, China. The GPV strain RC16 isolated was passaged and identified, whose full-length genome is 5,046 nucleotides long.

## GENOME ANNOUNCEMENT

Goose parvorirus (GPV) is the member of the *Dependoparvovirus* genus of the *Parvovirinae* subfamily within the *Parvoviridae* family. GPV is fatal for goslings, can cause enteritis and diarrhea, and is the etiological agent of Derzsy’s disease (1, 2). GPV is a single-stranded DNA virus without envelope proteins. The intact genome of GPV is about 5,100 nucleotides (nt) long (3) and contains two major open reading frames (ORFs) and inverted terminal repeats (ITRs) at both ends of the genome. The left ORF encodes the nonstructural protein required for both replication of the viral genome and regulation of capsid gene expression (4, 5), and the right ORF encodes the structural protein composed of a 3-capsid protein which has a common C-terminal region (3).

We isolated a goose parvovirus strain from the liver of an ill goose in 2016 at Rongchang, Chongqing, China, which we named goose parvovirus strain RC16. The clinical signs of the sampled goose were lethargy, ataxia, weight loss, palpebral swellings, dysphagia, and anorexia. The strain RC16 was serially passaged in 11-day-old embryonated duck eggs eight times, with mortality rates reaching 65%. Moreover, it also was serially passaged on primary duck embryo fibrolast (DEF) cells eight times, which can cause cytopathic effect (CPE). Six pairs of specific primers based on the sequence of the virulent strain YZ99-6 (GenBank accession number KC996730.1) were designed to amplify the six fragments of strain RC16, which were sequenced by TSINGKE Biological Technology (Chengdu, China), and then the full sequence was assembled by SeqMan. The full sequence of strain RC16 is 5,046 nt in length. Compared with the virulent strain LH (GenBank accession number KM272560.1), the ITR of the goose parvovirus strain RC16 is 414 nt in length (6). The left ORF is 1,884 nt in length and encodes the nonstructural protein composed of 627 amino acids. The right ORF is 2,199 nt in length and encodes the structural protein composed of 732 amino acids.

After phylogenetic analysis, strain RC16 was found to be closer to the virulent strain YZ99-6, the virulent strain LH, and the GPV WX (GenBank accession number KR091959.1) than to the GPV vaccine strain SYG61v (GenBank accession number KC996729.1), the GPV virulent B strain (GenBank accession number U25749.1), and GPV strain Y (GenBank accession number KC178571.1). Compared with the genome sequences of the virulent strain YZ99-6, the virulent strain LH, and GPV WX, strain RC16 shows high identity (99.11%, 99.03%, and 98.69%, respectively) at the nucleotide level. However, strain RC16 has slightly low identity with the GPV vaccine strain SYG61v, the GPV virulent B strain, and GPV strain Y, at 93.61%, 96.96%, and 97.59%, respectively.

Herein, we report the identification and complete genome sequence of the goose parvovirus strain RC16 isolated from an ill goose. It is useful for us to further understand the epidemiology and evolution of GPV.

### Accession number(s).

The genome sequence of the goose parvovirus strain RC16 has been deposited in GenBank under the accession no. KY475562.
